# Bradyphrenia and Tachyphrenia in Idiopathic Parkinsonism Appear, in Part, Iatrogenic: An Observational Study with Systematic Review Background

**DOI:** 10.3390/jcm12206499

**Published:** 2023-10-12

**Authors:** Wenjing Wang, Kieran Baker, Chianna Umamahesan, Steven Gilmour, André Charlett, David Taylor, Allan H. Young, R. John Dobbs, Sylvia M. Dobbs

**Affiliations:** 1 South London and Maudsley NHS Foundation Trust, London SE5 8AB, UKchianna.umamahesan@kcl.ac.uk (C.U.); david.taylor@slam.nhs.uk (D.T.); allan.young@kcl.ac.uk (A.H.Y.); 2 Institute of Psychiatry, Psychology & Neuroscience, King’s College London, London SE5 8AF, UK; 3 Department of Mathematics, King’s College London, London WC2R 2LS, UKsteven.gilmour@kcl.ac.uk (S.G.); 4 Institute of Pharmaceutical Science, King’s College London, London SE1 9NH, UK; andre.charlett@ukhsa.gov.uk; 5 Statistics, Modelling and Economics, UK Health Security Agency, London NW9 5EQ, UK; 6 Department of Gastroenterology, King’s College Hospital, London SE5 9RS, UK

**Keywords:** bradyphrenia, Parkinson’s disease, iatrogenic, levodopa, anti-muscarinic

## Abstract

We question whether bradyphrenia, slowing of cognitive processing not explained by depression or a global cognitive assessment, is a nosological entity in idiopathic parkinsonism (IP). The time taken to break contact of an index finger with a touch-sensitive plate was measured, with and without a warning in the alerting signal as to which side the imperative would indicate, in 77 people diagnosed with IP and in 124 people without an IP diagnosis. The ability to utilise a warning, measured by the difference between log_e_-transformed reaction times (unwarned minus warned), was termed ‘cognitive efficiency’. It was approximately normally distributed. A questionnaire on self- and partner perception of proband’s bradyphrenia was applied. A multivariable model showed that those prescribed levodopa were less cognitively efficient (mean −5.2 (CI −9.5, −1.0)% per 300 mg/day, *p* = 0.02), but those prescribed the anti-muscarinic trihexyphenidyl were more efficient (14.7 (0.2, 31.3)% per 4 mg/day, *p* < 0.05) and those prescribed monoamine oxidase-B inhibitor (MAOBI) tended to be more efficient (8.3 (0.0, 17.4)%, *p* = 0.07). The variance in efficiency was greater within IP (F-test, *p* = 0.01 adjusted for any demographic covariates: coefficient of variation, with and without IP, 0.68 and 0.46, respectively), but not so after adjustment for anti-parkinsonian medication (*p* = 0.13: coefficient of variation 0.62). The within-participant follow-up time, a median of 4.8 (interquartile range 3.1, 5.5) years (101 participants), did not influence efficiency, irrespective of IP status. Perception of bradyphrenia did not usefully predict efficiency. We conclude that both bradyphrenia and ‘tachyphrenia’ in IP appear to have iatrogenic components, of clinically important size, related to the dose of antiparkinsonian medication. Levodopa is the most commonly prescribed first-line medication: co-prescribing a MAOBI may circumvent its associated bradyphrenia. The previously reported greater efficiency associated with (low-dose) anti-muscarinic was confirmed.

## 1. Introduction

The term “bradyphrenia” was introduced in 1922 by Naville [1] to describe the slowing of cognitive processing associated with parkinsonism consequent on pandemic encephalitis lethargica. It was almost pathognomonic of this condition but had been noted previously in classical idiopathic parkinsonism (IP). 

Using the straightforward objective definition of slowing of cognitive processing, unexplained by depression or a global cognitive assessment, over and above any ageing effect, and irrespective of bradykinesia, we found bradyphrenia to be an entity in IP [2,3]. Since, overall, anti-parkinsonian medicines appeared to speed up cognitive processing [2], we put bradyphrenia forward (1993) as being nosological (i.e. classifiable as part of the disease process) [2,3]. We now revisit the extent and directionality of the iatrogenic influence (i.e. due to medical activity) on our original measure of cognitive processing time [2], uniquely employing a multivariable analysis of the effect of concurrent medicinal classes and their dosages. The contribution of this observational study to existing knowledge on the effects of anti-parkinsonian medicines on cognitive processing time is set in the context of a systematic review.

We incorporate a wider perspective on the potential consequences of bradyphrenia in a screening questionnaire, cross-referencing the proband’s perception against that of their spouse/life partner/carer. We examine whether perceptions are, indeed, associated with objectively measured cognitive slowing. This wider perspective encompasses deficits in attention and multi-tasking and difficulties in sequencing and planning. Deterioration in walking performance, as the demands of conversation intrude [4], may be an important everyday consequence. If bradyphrenia is iatrogenic, these consequences may be remediable. Masking bradyphrenia, by trading-off cognitive processing speed against accuracy, may no longer be called upon.

It is interesting to note that, in IP, psychosis appeared relatively rare before dopaminergic therapies [5], but, of course, there were no corresponding data quantifying bradyphrenia. Currently, bradyphrenia may be underacknowledged because of its covert nature, and clinicians being focused on the motor outcome or too ready to dismiss bradyphrenia as inevitable. We suggest that a better understanding of its place in the evolutionary pathways of IP is important. Indeed, might it, whether nosological or iatrogenic, be a harbinger of dementia?

## 2. Materials and Methods

### 2.1. Participants

This observational study was set in a National Research Clinic with a focus on quantifying disease facets to define temporal and interventional change. People with diagnosed IP were invited to volunteer. Inclusion was according to UK Brain Bank Criteria [6]: diagnosis of Parkinsonian syndrome (bradykinesia and at least one of the following—muscular rigidity, rest tremor and postural instability), with at least three supportive criteria (from unilateral onset, rest tremor present, progressive disorder, persistent asymmetry affecting the side of onset more, and clinical course of 10 years or more), after excluding causes of secondary parkinsonism. Evidence of response to levodopa was not required. Probands’ cohabiting life partners were invited to enlist. ‘Controls-proper’ neither had diagnosed IP nor resided with anyone who did. The study was approved by King’s College London Research Ethics Committee, with participants giving written informed consent.

Table 1 contrasts the demographic characteristics, medication and psychomotor/psychometric measures, including unwarned and warned reaction times and scores for perception of bradyphrenia, in 77 participants with diagnosed IP and the ‘remainder’, 124 without IP (including 58 partners of IP probands and 19 with a known family history of IP). Seven participants (six with IP and one without) had been excluded because their mini-mental state examination (MMSE) was less than 27/30 (an upper limit commonly used when targeting subjects with minimal cognitive impairment or mild Alzheimer’s disease [7]).

### 2.2. Reaction Time Testing

As the etymology implies, a test of bradyphrenia should isolate cognitive speed. We measure the efficiency of cognitive processing objectively based on the amount of shortening of the reaction time in response to a warning signal. We minimise any influence of motor function using a motor task of breaking finger contact with a touch-sensitive plate. The times taken for predictable (with warning) and unpredictable (without warning) tasks are contrasted [2]. Subjects responded to a command on the computer screen, “GO LEFT” or “GO RIGHT”, by breaking contact of their corresponding index finger. Prior to the command, one of three possible warning signals appeared, “READY”, “READY LEFT” or “READY RIGHT”, which does not warn or warns whether the imperative will be to break contact on a specified side. The delay between the warning and the imperative signals was fixed at 2 s, as this was found to be more discriminant for IP than randomly varying between 1 and 3 s [2]. There were four possible combinations of test conditions (unwarned or warned, each with right or left). For each participant, random permuted blocks were used to allocate the order in which these combinations were presented. Participants performed four replicates of each combination for practice, 10 blocks in the ‘test proper’ where the geometric mean for each combination was taken as the estimate. Wrong responses, classified as lifting the wrong finger or an excessively fast response (‘jumping gun’ (<100 ms)) were rejected. The data were processed with an in-house ‘PD-Tool Box’ computer programme.

### 2.3. Efficiency of Response to Warning Signal 

**Figure 1:**
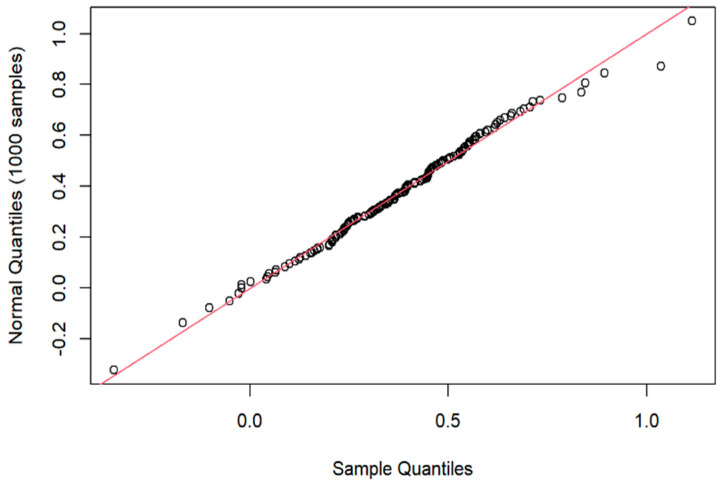
**Quantile–quantile plot comparing two probability distributions: quantiles of natural log ratio of reaction times (sample) plotted against those of a Gaussian distribution.** Points lie close to red line y = x, supporting assumption of normality.

‘Cognitive efficiency’, the efficiency of a response to a warning signal, was measured as the difference between the natural log of the unwarned reaction time and that of the warned reaction time, equivalent to the natural log of the ratio of reaction times [2]. This assumes that the motor component of the total reaction time is constant within all participant occasions: a criterion addressed by the motor task always simply being to break contact of a specified (right or left) index finger with its touch-sensitive plate (see Section 2.2). Figure 1 supports the assumption that cognitive efficiency is normally distributed.

### 2.4. Systematic Review on Bradyphrenia

#### 2.4.1. Development of Questionnaire 

The questionnaire is based on our systematic review to capture the wider usage of the term bradyphrenia in IP, with validation of participant experiences against objective measures. 

**Figure 2:**
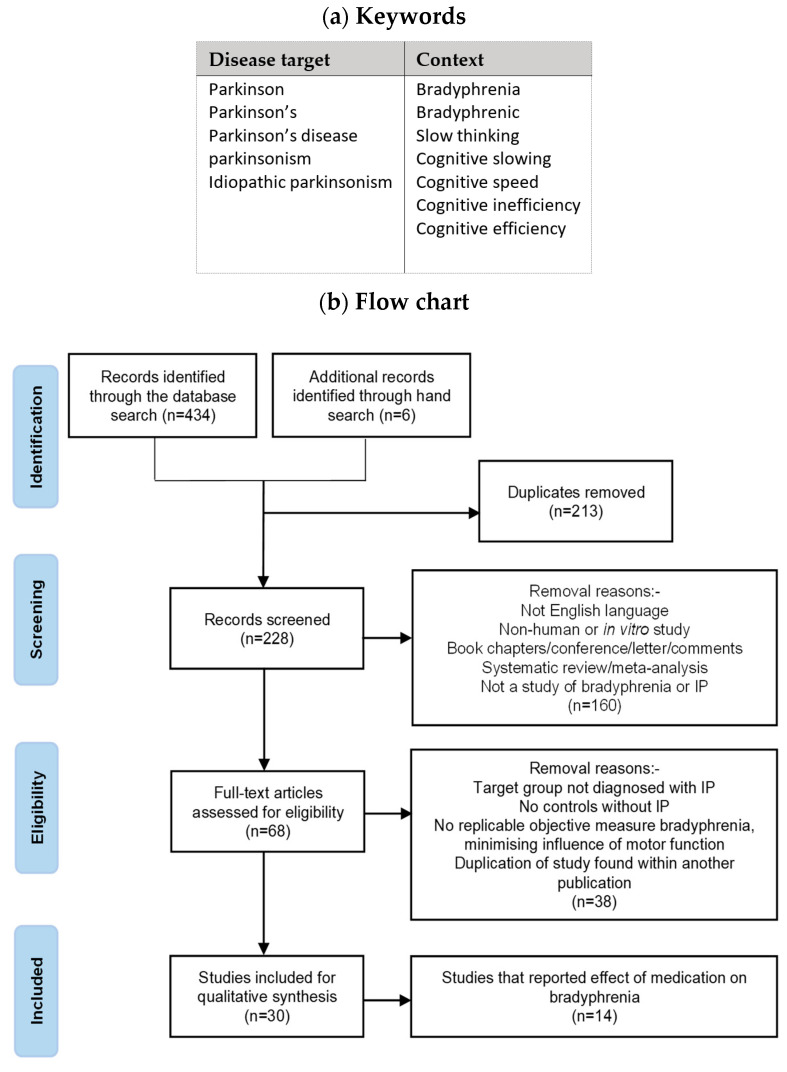

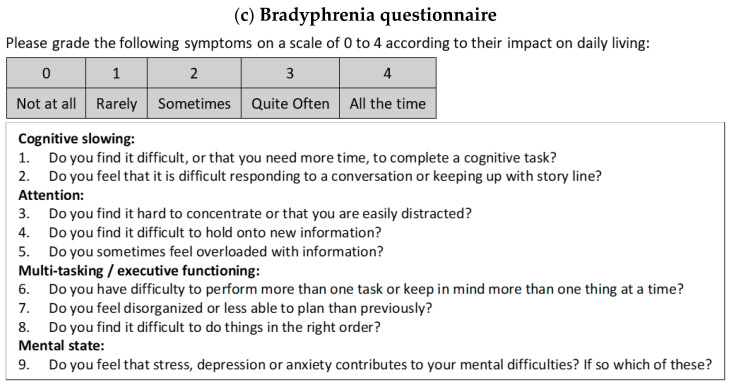
**Systematic review: (a) search terms, (b) flow chart based on PRISMA guidelines and (c) bradyphrenia questionnaire**. Manual search, in (**b**), results from references in relevant articles.

The search strategy is based on the ‘Condition, Context and Population’ framework [8]. Here, a population is the people with or without the target condition of IP, with its variable combination of defined signs, in the absence of a recognised cause. The context is bradyphrenia. The search terms based on disease target and context are shown in Figure 2a. Figure 2b gives the stages of the systematic review, identification, screening, eligibility assessment and the inclusion decision, in line with the ‘Preferred Reporting Items for Systematic Reviews and Meta-Analyses’ (PRISMA) guidelines [9]. The Ovid, Medline, Embase and PsycINFO databases were used to search for articles published between 1946 and 2023 in peer-reviewed scientific journals. Screening of sources, titles and nature of publication (by primary reviewer WW) excluded papers not in English and those without a translation into English; animal and cell studies; book chapters, conference abstracts, letters and comments; systematic reviews and meta-analyses; case histories; and not apparently being a study of bradyphrenia or one in IP. Eligible were studies obeying the inclusion criteria: having a target group with diagnosed IP (with no history of other neurological disease, dementia or affective disorders) and a comparator group without IP; replicable objective quantification of bradyphrenia (e.g. information processing speed or reaction time) with attention paid to minimising the influence of motor function (e.g. subtraction of time taken for a cognitively simpler task from that taken for a more complex task, where motor components are similar). The secondary reviewers (RJD and SMD) assessed any equivocal selections.

Figure 2c gives the resultant bradyphrenia questionnaire, tempering the need to encompass difficulties associated with bradyphrenia with that of being quick and easy to perform. Questions 1 and 2 relate to cognitive slowing, questions 3 to 5 relate to attention, questions 6 to 8 to multi-tasking and executive function, whilst question 9 addresses any effect of mental state on cognitive inefficiency. For IP probands, an additional ‘observer’ score was made in isolation by the corresponding spouse/partner/carer. The total score and responses to each question (graded as 0–4) were analysed.

#### 2.4.2. Assessment of Iatrogenicity

The selected studies were screened further (Figure 2b) to home-in on those reporting the effect of medication on cognitive processing time. The following information was extracted from each article: (i) citation; (ii) type of study: randomized or open; cross-sectional observational (cohort or case–control); within- and/or between-subject comparisons (within-dose interval fluctuations in medicinal effects, presence/absence of treatment or specified medication, and dose–response relationship); (iii) cohort size; (iv) methodology for measuring outcome; (v) clinical outcome(s).

### 2.5. Statistical Analysis

Two research questions were addressed: (**i**) Can any objective difference in cognitive efficiency according to IP status be explained by medication? The effects of explanatory variables on cognitive efficiency were investigated using multivariable linear regression models. Medicinal effects were examined using stepwise variable elimination, with model selection according to the Akaike information criterion [10]. All potential confounders (demographic, MMSE, and depression and anxiety scores) were retained in the models. Parameter estimates (confidence intervals) were exponentiated to be expressed in terms of relative change in cognitive efficiency. (**ii**) Is the total score, or subscores (five-point graded answer), of questionnaire on bradyphrenia perception associated with cognitive inefficiency? A linear mixed model was used with random participant intercepts, with the response variable being cognitive efficiency and the explanatory variables being individual questions and potential covariates. 

## 3. Results

### 3.1. Demographics 

**Figure 3:**
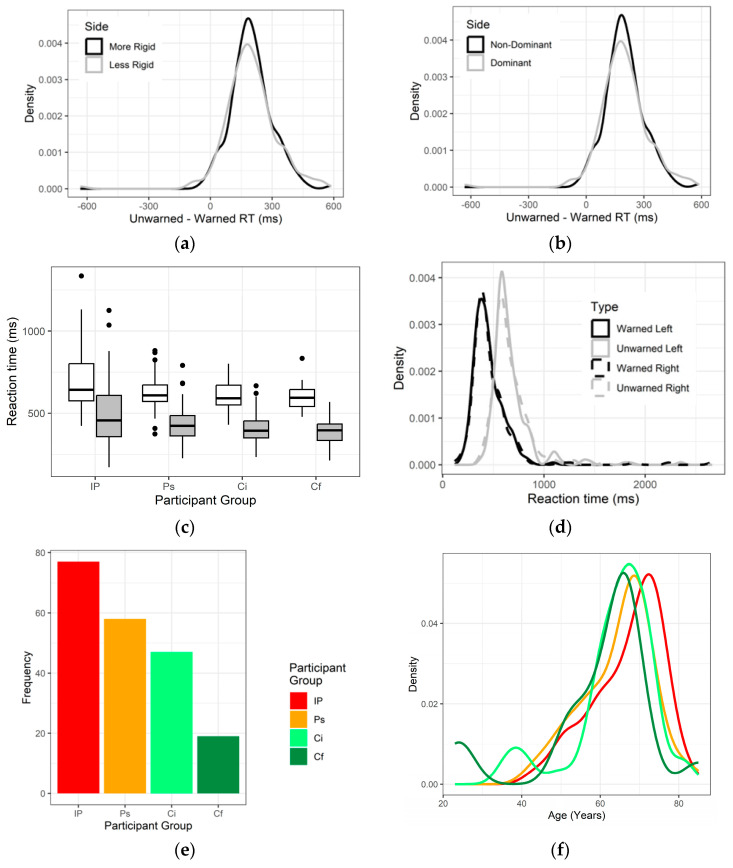

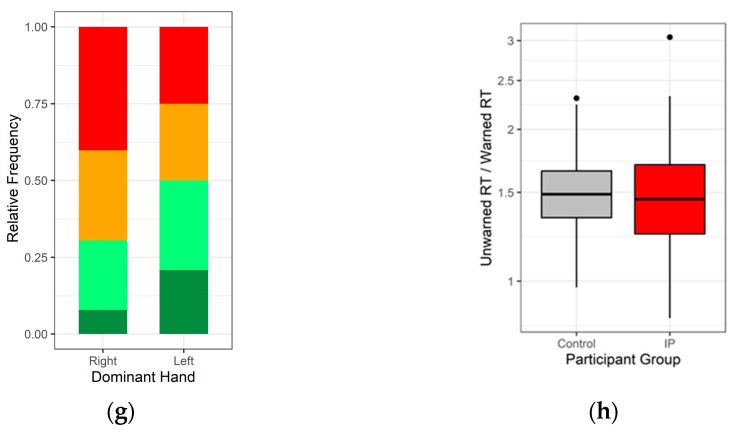
**Distribution of some key variables.** Probability density function, using bandwidth for optimal smoothing, for difference between unwarned and warned reaction time (RT) according to whether (**a**) more or less rigid side or (**b**) dominant or non-dominant hand used. (**c**) shows RT per participant group (P, with IP; Ps, life partner of IP proband; Ci, index control; Cf, control with family history of IP) according to whether alerting signal contains a warning (grey-filled box) or not (white): this is summarised for all participants, with reference to the side used, in (**d**). (**e**) gives participant numbers, (**f**) their age distribution and (**g**) relative frequency of hand dominance, by participant group. (**h**) shows ratio of unwarned to warned RT in those without IP (grey) compared with those with IP (red). N.B. Box and whisker plots in (**c**,**h**) show median (Q2), quartiles (Q3 and Q4), and maximum and minimum values, with outliers (Q2 ± 1.5 × (Q3 − Q1)) shown as filled circles.

Figure 3a–g show the distribution of cognitive efficiency and reaction time according to the test conditions and that of age and dominant hand according to the participant group. Shortening of reaction time, in response to a warning as to whether left or right index finger should break contact, was irrespective of side used, dominance of that side, or whether it was the more or less rigid. Indeed, these conditions were confounded: 89% of participants were right-dominant and 89% had greater resistance to passive arm movement on left. The reaction times for the left and right sides were averaged in further analysis.

### 3.2. Is Cognitive Efficiency Different between Those with and without Diagnosed IP?

Overall, age, gender, body mass index, MMSE score, and depression and anxiety ratings did not contribute to the multiple regression model for cognitive efficiency but were included in further modelling to adjust for any covariate effects in the presence of other variables. 

We considered whether the sub-groupings, life partners and controls with a family history of IP, could be pooled with the rest of those without diagnosed IP. There was, indeed, no significant difference in cognitive efficiency between these three subgroups (*p* = 0.5, Kruskal–Wallace test). Figure 3h shows that those with IP had a greater variation in the ratio of unwarned to warned reaction time than the pooled remainder of participants. This was formalized by comparing the variance, which measures variability from the mean (variance estimate in cognitive efficiency with and without IP 0.0503 and 0.0304, respectively, F-test *p* = 0.01). After adjustment for anti-parkinsonian medication, the variance in cognitive efficiency was not significantly different in IP (adjusted variance estimate 0.0412, *p* = 0.13). The coefficient of variation was, in IP, 0.679 before and 0.616 after adjustment, and that in the remainder was 0.458.

### 3.3. Effect of Medicines on Cognitive Efficiency 

A multivariable approach was essential given the frequent combined usage of anti-parkinsonian medicines. Variance inflation factors were less than 5 in all cases, indicating acceptably low multicollinearity [11]. None of the demographic covariates significantly contributed to explaining cognitive efficiency, before or whilst taking account of medicinal effects. Table 2 shows the binary predictors of cognitive efficiency in IP. This translates, in terms of dosage, to a deleterious effect of 5.2 (95% CI 1.0, 9.5)% on cognitive efficiency per 300 mg/day of levodopa and a beneficial effect of 14.7 (0.2, 31.3)% per 4 mg/day of the anti-muscarinic, trihexyphenidyl. The trend to benefit from the presence of a MAOBI was 8.3 (0.0, 17.4)%. We ask whether co-prescription of a MAOBI and/or COMTI alters the association of levodopa with bradyphrenia. There were 22 IP probands taking levodopa with a COMTI, a MAOBI or both and 18 taking levodopa without a COMTI or MAOBI. A MAOBI was taken without levodopa in 17. None, of course, were taking a COMTI without levodopa. No significant interaction between levodopa and COMTI and/or MAOBI on cognitive efficiency was found, but a clinically important effect modification cannot be completely excluded. Documented non-anti-parkinsonian medicines (laxatives, anti-depressants, anti-psychotics and NSAIDs) were not featured in the model. 

### 3.4. Effect of Medicines on Reaction Time

Reaction times are complex, including motor (here breaking finger contact) as well as cognitive efficiency components. In multivariable modelling, both unwarned and warned reaction time increased with the covariates age and depression, warned reaction time also increasing with weight: adjustment was made in the modelling of anti-parkinsonism medication effects (Table 3).

Reaction time was considerably worse on levodopa, with a numerically larger increment being seen in the warned than the unwarned reaction time, fitting with the decrease in cognitive efficiency (Section 3.3). Anti-muscarinic medication tended to be associated with a better warned reaction time (−78 (−172, 15) ms, *p* = 0.1) but had no significant effect on the unwarned, findings fitting with the benefit to cognitive efficiency. Both the unwarned and warned reaction times were faster in the presence of MAOBI medication. Anti-psychotic treatment was associated with markedly worse reaction time, confined to the unwarned. 

Our ‘cognitive efficiency’ outcome is a measure of the ability to make use of the warning in the alerting signal. It is important to validate the outcome according to this prerequisite (Section 2.3). Paradoxical effects were seen: Reaction times worsened on dopaminergic agonists, but here, the greater effect appeared to be on the unwarned reaction time, resulting in a numerical ‘improvement’ in cognitive efficiency (11.1 (−1.0, 24.1)% per 2.1 mg/day pramipexole equivalent). Similarly, anti-psychotics worsened the unwarned, relative to any effect on the warned, with ‘improvement’ in efficiency. Amantadine improved the unwarned reaction time, having no effect on the warned, resulting in a numerically large ‘worsening’ in cognitive efficiency (−11.3 (−22.9, 2.0)% per 100 mg/day). An explanation of the paradox might be medicinal effects on alertness, which can be overridden by the response to the prompt in test alerting signal. 

### 3.5. Longitudinal Follow-Up on Cognitive Efficiency

In IP, follow-up on cognitive efficiency was available in 101 participants (108 observations in 43 with IP and 58 without IP) over a median of 4.76 (interquartile range 3.06, 5.51) years. The time lapse from its first assessment had no effect on efficiency, irrespective of the presence of IP.

### 3.6. Bradyphrenia Questionnaire

The estimated within-participant standard deviation (1.43) suggested some variability in the responses across the questions. That is, they were not selecting the same grade (0 to 4) for each question. The total bradyphrenia score was not a significant predictor of cognitive efficiency. Table 4 shows that Question 1 (“Do you find it difficult, or that you need more time, to complete a cognitive task?”) was the only significant item in this respect (*p* = 0.01). Whilst Question 1 best fits with the purpose of the cognitive efficiency metric, the percent of variance it explained was negligible (R^2^ = 0.14). Over all questions, the odds ratio for the observer’s grading being higher than the proband’s self-grading was 1.73 (95% CI 1.35, 2.22) (*p* = 0.001). 

### 3.7. Systematic Review of Medicinal Effects on Bradyphrenia in IP

Table 5 classifies, according to design, the 14 retrieved studies of associations of anti-parkinsonian medication with bradyphrenia. 

Of the four studies comparing, within-participant, off-state (deprived of one or more doses of dopaminergic medication) and on-state (repleted with) [12,13,14,15], memory scanning speed in the on-state was slower in two studies [12,15] not different in one [14]. Task-set switching time was better in on-state in one study [13]. In only one study [12] was the on/off-state entirely attributable to levodopa. The assumption that all classes of dopaminergic therapy will have same directionality of effect may also have contributed to the lack of effect in two within-participant comparisons of being off- or on-maintenance therapy [16,17], added to which there was lack of [16] or inadequate [17] counterbalancing of treatment order. 

Regarding between-participant comparisons, two studies showed no dose–response relationship for levodopa on cognitive processing speed [2,18], but a univariable analysis of each anti-parkinsonian medication taken showed an improvement in cognitive efficiency on low-dose anti-cholinergic and on amantadine [2]. (Only the anticholinergic finding was replicated in the current multivariable analysis (Section 3.3).) Other analyses, where medicines were simply categorised as dopaminergic or cholinergic [19] or as dopaminergic or not [20], showed no association with bradyphrenia outcomes. Four studies [21,22,23,24], where the comparisons included an untreated subgroup, failed to distinguish any medicinal effect from disease progression. Eighteen-hour abstinence from anti-parkinsonian medication was associated with better task set switching than in a comparator group taking medication [13].

## 4. Discussion

Summarizing our findings, people with IP have greater variance in cognitive efficiency than controls. The bradyphrenia of IP appears to have a substantial iatrogenic component. Tachyphrenia (i.e. quickened cognitive processing), termed for the first time in IP, is associated with specific medication. Five-year follow-up in half of the cohort shows no change in cognitive efficiency, according to IP status, suggesting that any nosological component is very slow in progression if not static. Exploring candidate nosological drivers is outside the scope of this study, comprehending iatrogenic components taking precedence. However, we have previously found an association between the archetypical bowel dysfunction of IP and bradyphrenia [26], suggesting that bradyphrenia may also, in some part, be linked to the underlying disease process. Simply asking the probands directly whether they find it difficult to complete a cognitive task, or for their carers’ opinions, was of no value in predicting cognitive efficiency. Thus, objective measurement of bradyphrenia needs to be an integral part of pre-treatment and follow-up assessment. 

### 4.1. Set in the Context of Systematic Review of Medicinal Effects on Cognitive Processing Time

Our systematic review (Section 2.4.2) shows most of the eligible studies to be small, with analyses stopping at the level of treated/untreated or dopaminergic therapy presence/absence. No consistent picture emerges from these. Where there is a within-participant comparison off and on medication [16,17], a lack of, or inadequate, counterbalancing of treatment order makes interpretation difficult. There are two larger studies [2,22]. A between-participant univariable analysis on the presence/absence of specified medication [2] showed, as presented here, improved cognitive efficiency with trihexyphenidyl, but failed to detect any deficit with levodopa. No dose–response relationship to levodopa dose, duration of therapy or plasma concentration of levodopa or of its long t½metabolite, 3-O-methyldopa, was revealed. In the other larger study, comparison of treated and untreated groups [22] showed no differential effect.

### 4.2. Potential for influencing Research, Practice and Policy 

Multivariable modelling, as applied here to elucidate medicinal effects, has enormous potential in evaluating the impact of patterns of prescribing on cognitive efficiency. The current study is, to the best of our knowledge, the only exploration of the effect of anti-parkinsonian medicines on cognitive processing time using multivariable modelling. Its yield in terms of medicine-associated effects sets a template for the larger studies needed to understand the impact of current prescribing habits. In other words, our approach sets out to address complexity rather than to be confounded by it. Our systematic review reveals the paucity of informative interventional studies on cognitive processing time in IP: small study size and design deficits are not conducive to a clearcut conclusion.

Bradford Hill wrote “The clear dose-response curve admits of a simple explanation”, causality [27]. A current mainstay of anti-parkinsonian treatment, levodopa with a peripheral decarboxylase inhibitor, was associated with an, on average, 5% decrease in cognitive efficiency per a modest 300 mg total daily dose. There was an 8% trend to benefit in the presence of a MAOBI, statistical analysis indicating that the MAOBI effect on cognitive efficiency was quite apart from sparing levodopa dosage. Using low-dose trihexyphenidyl, we replicated our previous finding of an improved cognitive efficiency with anti-muscarinics [2]. In both studies, the antimuscarinic association was the most statistically significant medicinal effect seen on cognitive efficiency after correction for any demographic covariates. Moreover, the current size of the effect was 15% per 4 mg total daily dose of trihexyphenidyl. We emphasise that this benefit is in a group of IP probands naturally selected by tolerance of anti-muscarinic maintenance therapy. The benefit of anti-muscarinics on the motor facet of tremor is much quoted [28]. To this, we add their association with cognitive efficiency. 

At face value, repleting the dopamine stores by MAOBIs appears to have the opposite effect on cognitive efficiency to giving the transmitter’s precursor, levodopa. However, the effects of MAOBI are complex, with excess dietary amine absorption, excess biogenic amines (serotonin), decrease in metabolic end products (hydrogen peroxide, aldehyde and ammonium), and reduced pro-inflammatory cytokine and chemokine gene and protein expression [29]. Should future work demonstrate a beneficial effect on bradyphrenia of co-prescription of a COMTI with levodopa, this might be attributable to preventing accumulation of the long elimination half-time metabolite 3-O-methyldopa (associated with reduced stride length and abnormal foot-strike) [30]. 

### 4.3. Wider Implications

Medicines which provoke cognitive inefficiency may have a knock-on effect to encompass the wider ramifications of bradyphrenia. Difficulty in sequencing and multi-tasking may manifest as impaired verbal fluency and visual–spatial performance, e.g. [5,31]. The resultant difficulty in acquisition and retention of information may be wrongly perceived as memory loss. Intervention by removing the iatrogenic component early in the natural history is desirable, as are tailored objective measures of these wider potential outcomes. Whether iatrogenic cognitive inefficiency in IP is an avoidable forerunner to hallucinations and psychosis needs exploring: delay in processing may allow other thoughts to intervene. 

Furthermore, if the association of levodopa with cognitive slowing is shown to be causal, whether long-term levodopa usage increases the risk of dementia should be addressed. Precise physiological delivery of dopamine in time and space contrasts with flooding of the system by oral administration of its precursor. On the contrary, although here, anti-muscarinics are associated with a substantial improvement in cognitive efficiency, their association with dementia has been widely (but inconclusively) addressed. A systematic review with a meta-analysis showed an association between anticholinergic burden, accumulated for multiple health conditions, and dementia [32]. However, when confounding by indication was removed (excluding diagnoses of IP, multiple sclerosis, Huntington’s disease and Creutzfeldt–Jacob disease, where dementia risk could be better explained by indication than medicine), only 3 disparate classes of medicines, out of the 22 with an anticholinergic burden considered, were associated with dementia (i.e. antidepressants, antiepileptics and loop diuretics) [33]. Moreover, there was no clear relationship between anticholinergic potency and dementia risk, nor any effect of time gap between anticholinergic burden and dementia diagnosis (up to 20.5 years). The list of indicted medicines varies between studies [34,35]. Polypharmacy might add-up to a noxious anticholinergic burden, lack of adherence to regimen subtract (in real life, as little as 30% of prescribed medication is taken as intended [32]). In the face of uncertainty, randomised controlled trials are needed, for example comparing deprescribing of maintenance medicines to their continuation. In diseases with dementia overlap, such trials should consider other candidates, not just focus on anticholinergic burden.

### 4.4. Limitations

This is a hypothesis-generating study on whether any objective difference in cognitive efficiency, according to IP status, can be explained by medication. It provides data on which to base sample size calculations for future work. The current work can be regarded as a feasibility study for a more comprehensive approach to examining the associations of patterns of prescribing with cognitive processing time. Sample size may have constrained the associations found here. For example, testing for interactions in an observational study (e.g. whether the association of levodopa with cognitive inefficiency might be offset by co-prescription of a MAOBI or COMTI) will require a larger sample size than testing for the main effect. 

We stress that these are findings in UK residents attending a National Research Clinic and that they require confirmation in other cohorts. Nevertheless, the current cohort of consecutive recruits is clearly defined in Section 2.1 and Table 1, and none of the demographic covariates were confounders in the models for cognitive efficiency.

Within-participant cross-over studies, with exposure, withdrawal and re-exposure to the targeted medicines, will be needed to address causality. 

On a technical note, it is important to recognise that our ‘cognitive efficiency’ outcome requires scrutiny to ensure that the effects are, indeed, on the ability to make use of the warning (see Section 3.4). 

## 5. Conclusions

The pervasive use of levodopa may have unrecognised dangers. We demonstrate the relationship of bradyphrenia to levodopa, even at a low dosage, in a between-subject multivariable model. A similar effect might be seen within subjects, with bradyphrenia in the levodopa ‘on-state’ compared with the ‘off-state’: recognising such swings would be important to care. A practical question is whether the inefficiency can be combatted by co-prescribing a MAOBI or even a low-dose antimuscarinic. Whilst much belated work is done mapping the effect of prescribing patterns on cognitive processing time and ascertaining cause/effect relationships, there is a need for transparency in the face of uncertainty [32]. We advocate for monitoring cognitive efficiency by a simple reaction time test with and without a prompt in the alerting signal to break contact of finger with a touch-sensitive plate. Shared decision-making requires explanation of the potential risks and benefits of treatment options. Not ascribing the iatrogenic (potentially avoidable or rectifiable) as nosological (‘to be expected’) is the immediate aim. Inducing bradyphrenia may not just scale down functionality but have even more serious long-term consequences. Unravelling any aetiopathogenic contributors remains on the agenda. 

## Figures and Tables

**Table 1:** **Participant characteristics at first visit**.

Characteristics	Median (Lower, Upper Quartile) * at First Assessment	
	IP (N = 77)	Remainder (N = 124)
**Demographic**		
Age (years)	69 (61, 74)	66 (59, 70)
Sex (male)	57%	40%
Height (cm)	170 (160, 177)	169 (162, 178)
Weight (kg)	72 (63, 84)	71 (63, 82)
Body mass index (kg/m^2^)	24.4 (20.5, 28.9)	22.3 (19.6, 26.9)
Dominant hand (right)	92%	85%
Time since diagnosis (years)	5 (2, 10)	-
**Medication**: (total daily dose): % receiving		
Anti-parkinsonian medication	74%	-
Levodopa dosage (mg)	300 (250, 413): 52%	-
Monoamine oxidase-B inhibitor	48%	-
Catechol-O-methyl-transferase inhibitor	13%	-
Dopaminergic agonist dosage (mg) ^†^	1.19 (0.71, 2.10): 47%	-
Amantadine dosage (mg)	100 (100, 100): 6%	-
Anti-muscarinic (trihexyphenidyl) dosage (mg)	4 (4, 6): 12%	-
Laxatives	52%	12%
Non-steroidal anti-inflammatory drug (NSAID) ^ⱡ^	9%	9%
Anti-depressant	4%	8%
Anti-psychotic	9%	0%
**Psychomotor and psychometric**		
Mini-mental state examination (maximum 30)	30 (29, 30)	30 (30, 30)
Depression score (63)	10 (6, 15)	4 (1, 10)
Anxiety score (63)	12 (6, 17)	5 (2, 8)
Average unwarned reaction time (ms) ^§^	643 (576, 801)	604 (555, 669)
Average warned reaction time (ms) ^§^	456 (358, 609)	411 (351, 465)
Bradyphrenia questionnaire score ^#^ (maximum 36)	13 (6, 19)	6 (3, 11)

* Exceptions labelled as percents. ^†^ Dosage expressed as pramipexole base equivalent (https://www.medicinescomplete.com/). ^ⱡ^ Aspirin not included when prescribed in low doses as anti-platelet treatment. ^§^ Over all conditions. ^#^ In a subset of 37 IP probands and 55 of the ‘Remainder’. N.B. Current tobacco smoking was previously associated with improved cognitive efficiency [2]: here, only five with IP and three without IP were current smokers.

**Table 2:** Multivariable model of association of medicines with cognitive efficiency in IP.

Binary Predictor	Effect of Presence on Cognitive Efficiency (%) (N = 77)	
	Mean (95% CI)	*p*-Value
Levodopa	−8.6 (−15.6, −2.0)	0.01
Anti-muscarinic	24.6 (7.3, 44.8)	0.005
Monoamine oxidase-B inhibitor	8.3 (0.0, 17.4)	0.065

**Table 3:** **Multivariable model of medicinal associations with unwarned and warned reaction times**.

Binary Predictor	Effect of Presence on Reaction Time (ms) * (n = 77)			
	Mean Unwarned (95% CI)	*p*-Value	Mean Warned (95% CI)	*p*-Value
Levodopa	108 (60,157)	0.001	133 (85, 182)	0.001
Dopaminergic agonist	109 (37, 182)	0.003	81 (6, 155)	0.03
Anti-psychotic ^†^	193 (49, 339)	0.009		
MAOBI ^ⱡ^	−130 (−199, −61)	0.001	−137 (−210, −63)	0.001
Amantadine ^†^	−118 (−235, −1.3)	0.05		

* Both reaction time models included adjustment for the covariates age, depression score and weight. ^†^ The model selection process excluded amantadine and anti-psychotic medication in the case of warned reaction time. (If retained, size of effect was 5 (−120, 130) and 92 (−64, 248), *p* = 0.9 and 0.2, respectively, with little effect on other medicinal components in the model). ^ⱡ^ Monoamine oxidase-B inhibitor.

**Table 4:** **Ordinal regression model of association of individual questions in bradyphrenia questionnaire with cognitive efficiency**.

Predictor Question *	Effect on Cognitive Efficiency Mean (95% CI)	*p*-Value
Q1	0.065 (0.015, 0.116)	0.01
Q2	−0.017 (−0.074, 0.041)	0.6
Q3	−0.004 (−0.061, 0.053)	0.9
Q4	−0.005 (−0.051, 0.042)	0.9
Q5	−0.001 (−0.51, 0.049)	0.96
Q6	−0.004 (−0.050, 0.041)	0.9
Q7	−0.033 (−0.079, 0.012)	0.2
Q8	0.025 (−0.024, 0.073)	0.3
Q9	−0.003 (−0.052, 0.047)	0.9

* Five-point graded answer. N.B. None of the demographic covariates (age, gender, body mass index, MMSE score, and depression and anxiety scores) contributed significantly.

**Table 5:** **Relationship of bradyphrenia to anti-parkinsonian medication in IP**.

	Total Daily Dose Levodopa * or Levodopa Equivalent ^†^ [and Other Medication]	Number Subjects	Primary Relevant Outcome Measures	Findings
**Within-subject comparisons**				
***on/off effect***				
Poewe et al. (1991) [12]	Mean levodopa 1211 (SD 395) mg [none other]	12	Memory scanning speed ^§^ (‘off-state’ measured first)	Speed slower in ‘on-state’ compared with off-state
Cools et al. (2001) [13]	[levodopa, dopaminergic agonist or selegiline]	15	Task-set switching	At a lower cognitive load, dopaminergic medication remedied impairment in switching between two tasks
Press et al. (2002) [14]	[9 receiving levodopa, 3 dopaminergic agonist, 3 anticholinergic, 1 amantadine, 1 tolcapone]	10	Memory scanning speed ^§^ (on- and off-states order counterbalanced)	No change in speed or accuracy with dopaminergic state
Poston et al. (2016) [15]	Mean ‘levodopa equivalent’ ^†^ 659 (SD 397) mg	24	Memory scanning speed ^§^ during functional MRI scanning (on- and off-states order counterbalance)	Speed slower in on-state, but accuracy unaffected. Putamen hyperactivation in ‘off-state’ (*cf* 23 controls), lost in on-state. Loss correlated with slower memory scanning.
***before and during de novo dopaminergic treatment***				
Rogers et al. (1987) [16]	[10 levodopa, 2 dopaminergic agonist, with anticholinergic stoppage in 1]	12	Digit symbol test, with correction for motor response time using test with lower cognitive load	No change after introduction
***off and on medication***				
Grande et al. (2006) [17]	[11 levodopa, 3 dopaminergic agonist]	14	Negative priming with slower response latency to cued than non-cued tests in IP (inadequate counterbalancing by treatment order)	No difference according to medication status
**Between-subject comparisons**				
***dose–response***				
Dobbs et al. (1993) [2]	Median 500 (interquartile range 300, 600) [all on levodopa]	81	Ratio unwarned to warned RT ^ⱡ^	No effect on efficiency (dose, duration of therapy, plasma concentration during ‘therapeutic window’ or of long t½ metabolite 3-O-methyldopa)
Russ and Seger (1995) [18]	Grand mean 550 mg	58	Memory scanning ^§^ (28 all on levodopa) and visual discrimination (30, 21 of whom on levodopa) speed	Difference in speed between most and least complex test unrelated to levodopa dose
***presence/absence-specified medication (univariate analysis)***				
Wilson et al. (1980) [19]	[divided into taking dopaminergic or cholinergic medication, plus 1 untreated]	20	Memory scanning speed ^§^	No difference by taking dopaminergic or cholinergic medication
Dobbs et al. (1993) [2]	Median 500 (interquartile range 300, 600) [81 levodopa, 10 dopaminergic agonist, 21 selegiline, 5 amantadine, 21 anti-cholinergic]	103	Ratio unwarned to warned RT ^ⱡ^	No effect on cognitive efficiency of levodopa, but improved with anticholinergic and with amantadine
Arroyo et al. (2021) [20]	Mean ‘levodopa equivalent’ ^†^ 697 (SD 425) mg	48	Choice reaction time adjusted for simple reaction time	Dopaminergic medication dosage not correlated with cognitive processing time
***untreated and treated groups***				
Zimmermann et al. (1992) [21]	Maximum 500 mg [9 levodopa or dopaminergic agonist, 3 selegiline, 1 anti- cholinergic, 1 amantadine]	19	Choice RT ^ⱡ^ (with uncoded or coded imperative) minus simple RT ^ⱡ^	10 untreated recently diagnosed IP were impaired by coded imperative, but not by uncoded (compared with 17 controls): 9 treated impaired by both
Cooper et al. (1994) [22]	[levodopa, dopaminergic agonist, or anticholinergic monotherapy]	100	Choice reaction time corrected by subtraction of simple RT ^ⱡ^	37 untreated newly diagnosed IP, 26 on recently started monotherapy, and 37 chronically treated not differentially affected by medicinal treatment
Tachibana et al. (1997) [23] ^#^	[21 levodopa, 16 trihexyphenidyl, 7 dopaminergic agonist, 5 amantadine plus 6 untreated]	29	Latencies in EEG waveforms (elicited during a semantic discrimination task) known to be longer in PD than healthy controls	No significant effect of levodopa or trihexyphenidyl dosage
Dujardin et al. (2007) [24]	[not specified]	27	Paced auditory serial addition test	No difference between 13 treated and 14 early untreated.
***Maintenance and medication withdrawal groups***				
Cools et al. (2001) [13]	417 (SD 227) and 482 (337) mg, respectively [all levodopa, dopaminergic agonist and/or selegiline, 3 anticholinergics]	29	Task-set switching	Dopaminergic medication reduced impairment in switching between two tasks in 14 where medication ‘as normal’ compared with 15 where ≥18 h abstinence

* Levodopa given with a peripheral decarboxylase inhibitor. ^†^ Levodopa equivalent daily dose using conversion factors for other antiparkinsonian medication. ^ⱡ^ RT: reaction time. ^§^ Principle of Sternberg choice reaction time paradigm: mean RT (y-axis) was an approximately linearly increasing function of memory set size (x-axis), the intercept with y-axis representing motor component. ^#^ Reference [25] excluded from Table as expansion on previous 1997 paper [23] which is quoted in Table.

## Data Availability

The data are available upon reasonable request.
